# Infective Endocarditis Outcomes in Congestive Heart Failure Patients

**DOI:** 10.7759/cureus.71336

**Published:** 2024-10-12

**Authors:** Vivek Joseph Varughese, Chandler Richardson, Hata Mujadzic, Dominic Vacca, Prem Patel

**Affiliations:** 1 Internal Medicine, Prisma Health/University of South Carolina School of Medicine, Columbia, USA; 2 Medicine, Prisma Health/University of South Carolina School of Medicine, Columbia, USA

**Keywords:** all-cause mortality, congestive heart failure, continuous pacing, infective endocarditis, intermittent pacing

## Abstract

Background/objectives: The aim of this study is to analyze the difference in hospital outcomes for infective endocarditis (IE) admissions with and without comorbid congestive heart failure (CHF).

Methods: The National Inpatient Sample (NIS) was the database we used to find and sort patient data from inpatient hospitalizations. We then used logit probit regression to analyze the association between patients admitted for IE with and without CHF and their all-cause mortality rates. After collecting our sample population, we used propensity score matching to create our comparative cohorts of IE patients which were then analyzed to determine if there was an association between inpatient hospital outcomes and the presence of CHF.

Results: Among the patients admitted for IE, there was a statistically significant association between comorbid CHF diagnoses and an increase in all-cause mortality during hospital admission. The proportion of mortality was 7.8% during the hospitalization for IE admissions with comorbid CHF, while the proportion was 4.16% for the subset of IE admissions without comorbid CHF. The association of comorbid CHF and all-cause mortality during the hospital stay for IE admissions was significant with an odds ratio of 3.197 (2.089-4.891) after adjusting for age, sex, race, income, and other comorbidities. The association between the requirement for either temporary or continuous pacing during the hospital stay and co-existing CHF diagnosis in IE admissions was also significant with an odds ratio of 2.86 (1.720-4.761). Among the propensity-matched cohort analysis, there was a significantly higher proportion of IE admissions with co-existing CHF needing pacing (either temporary or continuous), 5.74% (3.96%-8.25%) compared to the IE cohort without comorbid CHF: 1.48% (0.71%-3.09%).

Conclusion: With the results of our analyses proving a significant association between the presence of CHF and adverse in-hospital outcomes in IE admissions, it calls for further prospective and real-world studies to analyze the finer aspects of the association. This becomes important for clinicians in stratifying at-risk patients for a higher level of care and prognostication aspects of the treatment. Our study results prove a causative association between the presence of CHF and heart block in IE admissions. This could be useful for clinicians in planning appropriate management plans in the event of initial signs of bradycardia and hypotension developing in this patient population.

## Introduction

Our study aims to explore the difference in hospital outcomes including all-cause mortality, length of stay, and total cost in adults >18 years old who are admitted with infective endocarditis (IE) and the presence or absence of congestive heart failure (CHF). First, we explored outcomes in IE patients with and without coexisting heart failure with consideration for confounding factors such as the presence of prosthetic valves, pacemakers, implantable cardiac defibrillators (ICDs), immunodeficiency, and drug abuse. Next, we explored the correlation between IE patients requiring temporary or continuous pacing during their admission and the presence of heart failure with the same outcomes as our end goal. All data was obtained from the National Inpatient Sample and analyzed using STATA 18 (StataCorp LLC, College Station, TX, USA). 

There have been several retrospective and randomized studies looking at outcomes of IE admissions when they have co-existing heart failure, and all of them did prove an association between poor outcomes in IE with heart failure. Our study looks at how the association applies to a larger population in real life, hence using the national in-patient sample for the study. Kiefer et al. did a prospective multicenter study analyzing associations between all-cause mortality and the need for cardiac surgery in IE patients complicated with CHF and found that 61.7% of the IE admissions with associated heart failure did undergo open heart surgery [1]. Pericàs et al. in their prospective randomized controlled trial proved a higher incidence of cardiogenic shock in patients having IE and co-existing CHF [2]. A sub-study, by Bohbot et al. of the European endocarditis registry, proved left-sided IE complicated by CHF was associated with a higher 30- and 90-day (about three months) all-cause mortality [3].

## Materials and methods

National Inpatient Sample (NIS) was used to obtain the population of adults, over the age of 18, admitted for the diagnosis of IE. International Classification of Diseases (ICD)-10 Procedure Coding System (PCS) code I330 (for acute and subacute endocarditis) was used to select the study population [4].

Among the IE admissions, patients with coexisting CHF were selected. The odds ratio associated with mortality, length of stay, and total charges were estimated. Confounding factors like drug abuse, valvular heart disease (mitral, aortic, and tricuspid), presence of pacemaker, ICD devices, heart transplant status, and mechanical prosthetic valves were used in the multivariate probit logistic regression as these factors have independent associations with both heart failure as well as outcomes in IE. Probit logistic regression and multivariate regression analysis were used to account for confounders while analyzing the association between all-cause mortality and the presence of heart failure in IE patients. Propensity score analysis and nearest neighbor matching were done to select matched cohorts from the IE population, with and without the diagnosis of CHF. The cohorts were matched exactly for age, race, household income, presence of ICD, presence of prosthetic valves, valvular heart diseases, intravenous (IV) drug abuse, and acquired immunodeficiency syndrome (AIDS). All cause-mortality and association with either continuous or intermittent pacing during the admission were analyzed. Sub-population of IE patients with heart transplant status was selected and in-hospital outcomes of mortality were compared with general IE outcomes. Lastly, ICD-10-PCS codes 5A1213Z (for intermittent cardiac pacing) and 5A1223Z (continuous cardiac pacing) were used to select IE admissions requiring cardiac pacing during the hospital stay. The association of temporary or continuous cardiac pacing with co-existing CHF was studied. ICD-10-PCS code Z952 was used to select subjects with the presence of prosthetic heart valves. ICD-10-PCS code trunks I34, I35, and I36 were used to select subjects with co-existing valvular heart diseases.

## Results

A total of 12869 patients were admitted for the main diagnosis of IE. Among them 58% (7464 admissions) of the admissions were male, 77.62% (9989 admissions) of the admissions were White, 10.23% (1317 admissions) Black, and 7.13% (917 admissions) were Hispanic. Of the admissions, 57.19% (7359 admissions) belonged to the lower 50 percentile median household income category. Crude mortality for IE admissions was 4.16%: 535 of the admissions for IE died during the admissions. Out of the total IE admissions, 4405 patients had a diagnosis of CHF.

When looking at our primary outcomes (Table 1), there was a statistically significant increase in all-cause mortality and mean total hospital charges in patients admitted for IE with comorbid CHF. Out of the total IE admission cohort, the prevalence of mortality was 7.83% (6.22%-9.80%)for admissions with a co-existing diagnosis of CHF (344 admissions out of the 4405 IE admissions with the diagnosis of CHF died during the admission), while the prevalence of all-cause mortality during the admission was 3.2% (2.7%- 4.6%) for the IE admission cohort without the diagnosis of CHF (270 out of the 8864 IE admissions without the diagnosis of CHF died during the admission). The mean of total hospital charges was higher for the IE admission cohort with CHF: 229334$ when compared to the total charges of 168273$ for the IE admission cohort without the diagnosis of CHF. We did not observe a statistically significant difference in the mean length of hospital stay when considering the presence or absence of CHF in IE admissions: 13.006 days (13.732-15.507) in the IE cohort without CHF versus 14.619 days (13.73-15.507) in the IE cohort with the diagnosis of CHF.

**Table 1:** Proportion of patients died during the admission for IE (with and without coexisting diagnosis of CHF) IE: infective endocarditis, CHF: congestive heart failure

	Proportion died	Mean length of stay	Mean of total hospital charges
IE admissions without coexisting diagnosis of CHF	.041 (.034-.050)	13.006 (13.732-15.507)	168273$ (158174.2$-178371.7$)
IE admissions with coexisting diagnosis of CHF	.078 (.062-.098)	14.619 (13.732-15.507)	229334.6$ (209011.1$-249658.2$)

When comparing the association of IE with and without CHF to all-cause mortality (Table 2), there was a statistically significant correlation between patients with IE and co-existing CHF and all-cause mortality that held true in both the unadjusted (odds ratio of 3.694) and adjusted (odds ratio of 3.197) odds ratios. The adjusted odds ratio accounted for patient age, race, sex, quarterly income, and associated comorbidities including the presence of valvular heart disease, prosthetic heart valves, ICDs, pacemaker, and IV drug use, and was significant with an odds ratio of 3.197 (2.089-4.891). There was no association between IE admissions without heart failure and all-cause mortality (p > 0.05).

**Table 2:** Association between all-cause mortality and coexisting CHF diagnosis in IE admissions IE: infective endocarditis, CHF: congestive heart failure, OR: odds ratio

Association with all-cause mortality	OR	P-value	Confidence interval
Odds of association between IE admissions without CHF and all-cause mortality	1.143	0.412	.829-1.575
IE admissions with CHF: unadjusted odds ratio	3.694	0.000	2.464-5.537
IE admissions with CHF: odds ratio adjusted for age, sex, race, and comorbidities	3.197	0.000	2.089-4.891

Complete heart block is a known complication of IE that can occur when an abscess forms around the conduction system of the heart. If this occurs during a patient's hospitalization, they will typically require either temporary or continuous cardiac pacing making this a significant complication of IE. Our study showed that patients who were admitted for IE with comorbid CHF had a statistically significant association with the need for cardiac pacing than those without CHF which remained consistent when adjusting for age, sex, race, quarterly income, and comorbidities. The odds of association of comorbid heart failure and requirement of either temporary or continuous pacing in IE admissions was 2.862 (1.720-4.761, p-value 0) (Table 3), while the odds of association between IE admission cohort without CHF and need for pacing was insignificant at 0.312.

**Table 3:** Association between CHF and the need for pacing in IE admissions IE: infective endocarditis, CHF: congestive heart failure, OR: odds ratio

	OR	P-value	Confidence interval
Association between Infective endocarditis admissions without CHF and need for pacing	.312	0.000	.193-.504
IE admissions with CHF: unadjusted odds ratio for association with need for pacing	3.200	0.000	1.982-5.167
IE admissions with CHF: odds ratio (adjusted for age, sex, race, and comorbidities) for association with need for pacing	2.862	0.000	1.720-4.761

Propensity score and nearest neighbor matching analysis

In order to better match outcomes among IE patients, propensity scoring, and nearest-neighbor matching were used to create a CHF-positive and CHF-negative cohort. Each cohort consisted of 470 patients who were matched for their age, race, AIDS presence, IV drug use, and the presence of either a pacemaker or ICD (Table 4).

**Table 4:** Propensity score matched cohorts from the IE admission population IE: infective endocarditis, ICD: implantable cardiac defibrillator, CHF: congestive heart failure, CHF+: cohort with congestive heart failure as co-diagnosis, CHF-: cohort without congestive heart failure as co-diagnosis

	CHF+ cohort	CHF- cohort
Total number	470	470
Mean age	58.131	58.131
Proportion with ICD	.0170	.0170
Proportion with prosthetic valve	1	1
Proportion with valvular heart disease	1	1
Proportion of drug users	.217	.217
Proportion died	.068 (.0485-.0947)	.0297 (.0176-.0497)
Mean length of stay	14.819 (13.685-15.953)	10.695 (9.516-11.874)
Mean of total charges	220319.8 (195823.6-244815.9)	127120.5 (105042.5-149198.6)
Proportion that needed pacing	.0574 (.039-.082)	.0148 (.007-.0309)

Using our matched cohorts, we compared our primary outcomes and the need for cardiac pacing during hospitalization. There was a statistically significant increase in hospital length of stay, total hospital charges, and the need for cardiac pacing among the CHF-positive cohort when compared to the CHF-negative cohort. There was no significant increase in all-cause mortality among CHF-positive patients. The proportion of all-cause mortality during the admission was 0.068 (0.0485-0.0947) for the CHF-positive cohort (32 admissions out of the 470 admission cohort) while it was 0.0297 (0.0176 - 0.0497) for the CHF-negative cohort (14 admissions out of the 470 admission cohort): the mortality was higher in the CHF cohort (6.8% vs 2.97%), but the difference did not reach statistical significance. The proportion that needed pacing in the CHF-positive cohort was .0574 (.039-.082), which was higher with statistical significance compared to the CHF-negative cohort with a proportion of .0148 (.007-.0309) that needed pacing. The mean length of hospital stay was 14.819 days (13.685-15.953) for the CHF-positive cohort, higher when compared to the CHF-negative cohort: 10.695 days (9.516-11.874). The mean of total charges in the CHF-positive cohort was 220319.8$, compared to 127120.5$ in the CHF-negative cohort.

## Discussion

The trends in the number of hospitalizations and the requirement for surgical interventions in the management of IE have been on the rise over the years [5]. Historically, IE was a disease that was limited to patient populations with rheumatic heart diseases, but with the growing prevalence of prosthetic valves, intracardiac devices, increase in the number of intravenous drug users, the epidemiology and prevalence of IE has changed to a point that suspicion for the condition should be high on the differential during the evaluation of unspecified fevers. In a 2013 meta-analysis done with 160 studies, the in-hospital mortality for IE admissions was at 25%, with 10.7% of the admissions having prosthetic valves, 14.6% of the admissions being IV drug users, with staph aureus being the causative organism isolated in 48% of the admissions [6]. Among the admissions, 3.8% were culture-negative. Among the admissions, 58% were males as per the meta-analysis. Looking at the epidemiology data from our analysis, we identified 12,869 patients admitted for the main diagnosis of IE using the National Inpatient Sample for the year 2019, with 58% of the admissions being males. This was concurrent with the findings of the meta-analysis. The presence of prosthetic heart valves is yet another risk factor for perivalvular abscess, and the growing trends in surgical aortic valve replacements (SAVR) and transcatheter aortic valve replacement (TAVR) procedures, would possibly explain the increasing trends in IE admissions over the years. In a real-world study by Pericàs et al., it was proved that the presence of valvular heart disease, i.e., mitral and aortic regurgitation was associated with a higher incidence of cardiogenic shock in IE admissions [2].

The primary goal of our article is to estimate how the presence of co-existing CHF changes outcomes in IE admissions. From the total IE admission pool, we identified 4405 admissions with co-existing diagnoses of heart failure (both systolic and diastolic heart failure). The prevalence of mortality during the admission for IE admissions without the co-existing diagnosis of CHF was 4.16%, while the prevalence for all-cause mortality during the admission was higher at 7.83% when there was a co-existing diagnosis of IE. This finding was proved with statistical significance. The mean of total hospital charges for IE admissions with a co-existing diagnosis of CHF was also significantly higher (Table 1).

As mentioned in the introduction, there have been several real-world studies proving worse outcomes for IE admissions when it is associated with CHF. In the second step of our study, we analyzed the association between diagnosis of CHF and all-cause mortality in IE admissions: we used logit probit regression to analyze the association. Choosing the comorbid conditions that were used in the logistic regression, we chose the presence of prosthetic heart valves, valvular heart diseases, and intravenous drug abuse as these were factors associated with worsened outcomes in IE admissions (as per multiple real-world studies and meta-analyses and has independent association with worse outcomes in CHF). Even after accounting for the comorbid conditions, the odds of association between the presence of CHF and all-cause mortality was high at 3.1972 (Table 2). Analyzing the association in IE admissions without a diagnosis of CHF, there were no significant associations seen. These findings are consistent with previously conducted retrospective analyses. Matsuura et al. did a retrospective real-world analysis involving 470 patients, and early surgical intervention proved to have better outcomes in IE admissions with heart failure refractory to medical management than delayed surgery [7]. In another real-world retrospective analysis by Jaffe et al., the presence of comorbid systolic heart failure is associated with higher chances of perivalvular abscess formation and heart block in IE admissions [8]. A real-world study by Pericàs et al. [2] looked at 4856 IE admissions of which 34% had a co-existing diagnosis of heart failure and 5% of them went into cardiogenic shock. Jamal et al. did a retrospective study on outcomes of complete heart block complicating IE admissions [9]. It proved that when there is a co-existing heart block complicating IE, there is a higher incidence of cardiogenic shock, acute kidney injury, and the requirement for a permanent pacemaker. Additionally, co-existing heart block was associated with a higher incidence of aortic and mitral valve replacements in IE admissions, and it also increased the total charges and the mean length of hospital stay. Looking through the literature, there have been several case reports showing how IE is associated with complete heart block, both transient and ones requiring a pacemaker. Although rare, right-sided endocarditis involving the triangle of Koch may present with conduction disease due to local inflammation and mechanical compression. Conduction disease associated with right-sided disease appears to be readily reversible with medical therapy and temporary device support may be appropriate in the acute setting. There have also been case reports describing triple valve endocarditis presenting with a complete heart block, and aortic root abscess with distal embolization [10]. Patel et al. have described a case where tricuspid valve endocarditis presents with a complete heart block [11]. There are cases of entire valvular occlusion related to IE in the presence of CHF [12], and multiple case reports on how IE after TAVR/SAVR procedures are associated with complete heart block [13,14]. Jiang X et al. did a single-center observational study on the outcomes of valvular surgery for mitral valve endocarditis and observed inferior outcomes with coexistent heart failure [15]. Summers et al. did a large-scale study using the TAVR registry in 2019, that proved that even though rare, the presence of perivalvular abscess associated with TAVR procedures can be extremely fatal [16]. Based on our analysis, there was a statistically significant association between the presence of heart failure in IE and the requirement of either temporary or continuous pacing. In the analyses of the association between the need for either continuous or temporary pacing and the presence of heart failure in IE admissions, significant results were seen. Like in the association with all-cause mortality, the regression analysis included comorbidities like the presence of prosthetic valves, valvular heart disease, IV drug abuse, and the presence of ICD/pacemakers. Our propensity-matched cohort analysis proved the causative association between the presence of heart failure and the requirement of pacing during the admission, and also total hospital charges and length of stay during the admission (Table 4). The causative relationship between comorbid heart failure in IE and all-cause mortality did not reach statistical significance in the propensity-matched cohort analyses.

Limitations of the study

The severity of heart failure, stratification between systolic and diastolic heart failure, and related outcomes were not done. Also, due to the limitations of the nature of the study being retrospective, outcome differences based on patients who are on GDMT and not on optimal medical therapy could not be analyzed. The National Inpatient Sample is a database with inherent limitations, and all the analyses based on the database are based on the assumption that there was accurate coding for each of the diagnoses and the comorbidities, which may not hold true in the real-world setting. Due to the nature of the database and associated limitations, differences in outcomes of left-sided vs right-sided endocarditis could not be analyzed. Nor was it feasible to analyze the differences in outcomes based on the causative organism.

However, this study provides valuable insights into the association between heart failure and outcomes in IE admissions. This association can inform clinicians about potential complications and prognostic indicators in patients with IE and heart failure. The use of propensity-matched cohort analysis strengthens the causal inference between the presence of heart failure and specific outcomes, such as the requirement for pacing, total hospital charges, and length of stay. Despite limitations, this study demonstrates the utility of real-world data from the National Inpatient Sample. This database allows for large-scale analyses that reflect patient populations in clinical practice, offering insights that may guide decision-making and resource allocation. Lastly, by highlighting the association between heart failure and outcomes in IE admissions, this study contributes to clinical knowledge and may prompt further investigation into tailored management strategies for patients with both conditions. Since clear inclusion criteria could not be applied for patient selection, the patient cohort we have will have questionable generalizability to the real-world scenario. Also, socioeconomic and demographic factors that could play a role in the outcomes of the study could not be addressed because of the nature of the database.

## Conclusions

We discovered that there is a significant correlation between an increase in all-cause mortality, mean length of hospital stay, total hospital charges, and the need for temporary or continuous cardiac pacing among IE admissions with comorbid CHF. This becomes crucial in the inpatient management of IE patients when there is comorbid CHF present, in terms of timely consultations and preparations for pacing in the at-risk population. We also noted that patients admitted for IE who had an ICD or pacemaker prior to hospitalization did not have a statistically significant association with an increase in all-cause mortality or hospital stay. 

One of the benefits of our study is the increased generalizability of the data due to the patient population studied compared to previous studies. There is still a need for ongoing research on the correlation between these two conditions, but it is also imperative to explore how this correlation affects patient care and to determine how best to care for these patients knowing that they are at an increased risk of several adverse outcomes.
